# Understanding the Multitarget Pharmacological Mechanism of the Traditional Mongolian Common Herb Pair GuangZao-RouDouKou Acting on Coronary Heart Disease Based on a Bioinformatics Approach

**DOI:** 10.1155/2018/7956503

**Published:** 2018-11-11

**Authors:** Jingkun Lu, Yuchong Hu, Lechun Wang, Yuewu Wang, Shengsang Na, Jian Wang, Yuhui Shun, Xiaoqin Wang, Peifeng Xue, Pengwei Zhao, Liping Su

**Affiliations:** ^1^School of Basic Medicine, Inner Mongolia Medical University, Huhehot, China; ^2^Inner Mongolia Autonomous Region People's Hospital, Huhehot, China; ^3^Pharmacy, Beijing United Family Hospital, Beijing, China; ^4^Center for New Drug Safety Evaluation and Research, Inner Mongolia Medical University, Huhehot, China; ^5^Institute of Mongolian Medicine, Inner Mongolia Medical University, Huhehot, China; ^6^School of Pharmaceutical Engineering, Shenyang Pharmaceutical University, Shenyang, China; ^7^School of Pharmacy, Inner Mongolia Medical University, Huhehot, China

## Abstract

GuangZao and RouDouKou (*Fructus Choerospondiatis* and* Nutmeg*, FCN) are one of the most common herb pairs in traditional Mongolian medicine for the treatment of coronary heart disease (CHD). However, evidence for the protective effect of FCN is limited, and its underlying mechanism of action remains unclear. The present study employed a network pharmacology approach to identify the potentially active ingredients and synergistic effects of the herb pair FCN as traditional Mongolian medicine. We predicted the targets of all available FCN ingredients with PharmMapper, SWISS, and SuperPred Server and clustered CHD-related targets from the DrugBank and the OMIM database. We also evaluated the links between herbal ingredients and pharmacological actions to explore the potential mechanism of action of FCN. We found that FCN targets a network of CHD-related key processes, including stress responses, cell adhesion and connections, angiogenesis, cell apoptosis and necrosis, the endocrine system, inflammatory and immune responses, and other biological processes. To confirm the predicted results, we investigated the protective effect of FCN on isoproterenol- (ISO-) induced myocardial ischemia in rats. Pathological assessment indicated that FCN inhibits apoptosis and inflammatory responses involving the myocardium. Quantitative real-time polymerase chain reaction (qRT-PCR) and western blotting analyses demonstrated the therapeutic effects of FCN on ISO-induced myocardial ischemia rats, possibly via regulating stress and inflammatory responses and inhibiting cardiomyocyte apoptosis. The findings of the present study indicate that bioinformatics combined with experimental verification provide a credible and objective method to elucidate the complex multitarget mechanism of action of FCN.

## 1. Introduction

Coronary heart disease (CHD) remains the leading cause of death in adults worldwide. CHD is a complex multifaceted condition. The main causes of CHD include myocardial ischemia, hypoxia, necrosis, and coronary atherosclerosis [1, 2]. Because CHD is the final manifestation of various pathological insults, there is a strong demand to find safe and efficacious multitargets products to combat this health epidemic. Traditional Mongolian medicine has been used to prevent and treat cardiovascular disease for many years using various herbal formulas and has lately been the subject of formal clinical studies. Herb pairs have been considered the halfway point between single herbs and herbal formulas, and typical herb pairs display basic synergistic effects of two kinds of herbs that have often been used in various herbal formulas with similar signs and symptoms. The GuangZao and RouDouKou (*Fructus Choerospondiatis* and* Nutmeg*, FCN) herb pair is commonly used in the treatment of CHD in traditional Mongolian medicine [3, 4]. The present study aimed to evaluate this herb pair as a potential CHD treatment regimen.

In traditional medicine, single herbs, herb pairs, and herbal formulas are treated as single units, and these are composed of diverse, complex components. Their pharmacological effects are generally due to various active monomers that involve multiple pathways and targets. Numerous studies have focused on the mechanism of action of various active monomers in herbs; however, these active monomers apparently have significantly different characteristics. The advent of systems biology and network pharmacology offers a novel opportunity to assess traditional medicine from the perspective of holistic regulation [5, 6].

**Figure 1:**
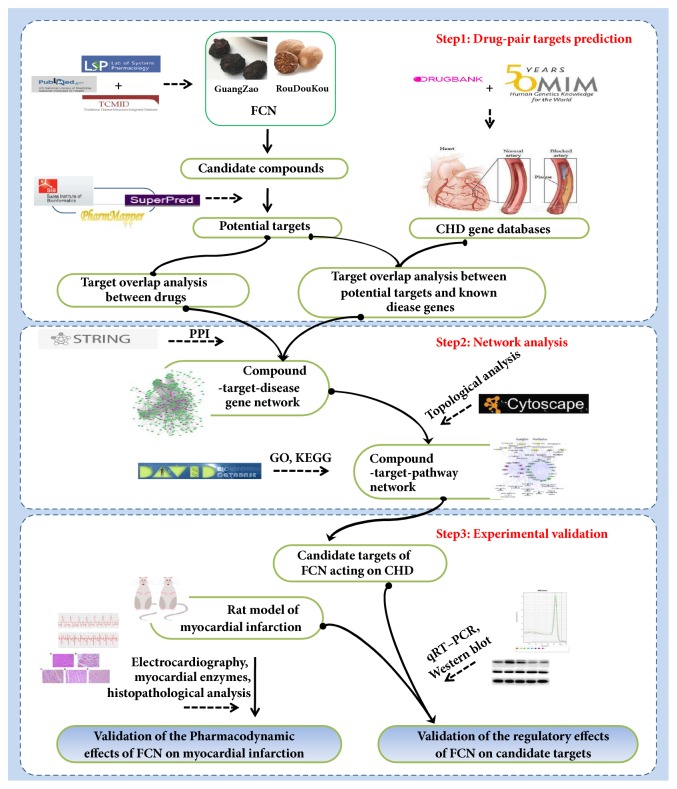
The schematic representation of our method involving bioinformatics combined with experimental verification for uncovering the mechanism of action of the FCN herb pair for the treatment of coronary heart disease (CHD).

The present study was designed to investigate the anti-CHD action of FCN based on network pharmacology, which encompasses identification of the components of FCN, CHD gene prediction-based data mining, drug target prediction, protein-protein interactions (PPI), drug-gene-disease comodule association, drug network analysis, and synergistic ingredients combination screening. Finally, an acute myocardial ischemia (AMI) experiment was performed on rats to confirm the mechanism of action of the FCN herb pair on AMI, including its targets and pathways. A schematic representation of our method is shown in Figure 1.

## 2. Materials and Methods

### 2.1. Computational Prediction of Anti-CHD Ingredients of FCN Using Network Target Analysis

#### 2.1.1. Data Collection

A total of 114 chemical ingredients and metabolites of GuangZao and RouDouKou in blood were obtained from the literature [7–26] and the Traditional Chinese Medicine Integrated Database (TCMID, http://www.megabionet.org/tcmid/). The chemical structures of the FCN ingredients were drawn with ChemBioDraw13.0. Prescreening of drug-likeness properties (DL) and oral bioavailability (OB) was performed using TCMSP (http://lsp.nwu.edu.cn/tcmsp.php) [27] to identify the active compounds of FCN. The chemical information (structure, canonical name, SMILES number, CAS number, and CID number) that was employed for computational analysis was downloaded from the PubChem Compound database (http://pubchem.ncbi.nlm.nih.gov/). A data set of CHD-related genes was retrieved from OMIM (http://www.omim.org/), which contains 958 CHD-related genes. In addition, the drug targets of 254 FDA-approved anti-CHD drugs were obtained from DrugBank databases.

#### 2.1.2. Prediction of Herbal Ingredient Targets

PharmMapper (http://lilab.ecust.edu.cn/pharmmapper/, version 2017), SWISS (http://www.swisstargetprediction.ch/, version 2013), and SuperPred (http://prediction.charite.de/index.php?site=home, version 2013) were used to predict the targets of FCN's candidate compounds. PharmMapper Server is designed to identify potential target candidates of small molecules using a pharmacophore mapping approach. SWISS and SuperPred are based on the principle that chemicals with similar structures have similar functions, and a 2D or 3D similarity search. The predicted targets that had normal fit scores ≥ 0.7 in PharmMapper, probability score ≥ 0.7 in SWISS, or known ligand-target in SuperPred were selected as target profiles for each molecule.

#### 2.1.3. Biological Function and Disease Ontology Enrichment Analysis of Target Profiles

The predicted targets of FCN ingredients were mapped with CHD-related genes obtained from OMIM and DrugBank databases. PPIs were assessed using STRING (https://string-db.org/, 2017) with minimum required interaction score ≥ 0.7 (high confidence). For identification and characterization of the mechanism of action of FCN, DAVID (https://david.ncifcrf.gov/, version 2013) was used for clustering analysis of the targets of the FCN ingredients. The results were then submitted for GO and KEGG pathway analyses, and terms with a* P *value of <0.01 were selected for functional annotation (both were corrected using the Benjamini method).

#### 2.1.4. Network Construction

Based on these results, the compound-target-pathway network was constructed using Cytoscape 3.5.1 software. Compounds were connected to their potential targets and genes statistically significantly linked to those targets via PPIs. An edge was added between a target and a pathway if this target gene was in this pathway. Important topological parameters in this network, including* Degree, Betweenness*, and* Closeness*, were analyzed.

### 2.2. Pharmacodynamic Study of FCN and Experimental Validation for Computational Prediction Results in a Rat Model of Isoproterenol- (ISO-) Induced Myocardial Infarction

#### 2.2.1. Drugs and Reagents

GuangZao and RouDouKou were purchased from Huhehaote Medicinal Company, (Huhehaote, China). The FCN herb pair (GuangZao and RouDouKou) is prepared at a ratio of 1:1. A TRIzol reagent Kit, SYBR® Premix Ex Taq™ II enzyme kit, and PrimeScript™RT Master Mix RT were purchased from Takara Bio, Inc. (Dalian, China), and a Total RNA Extraction Kit was purchased from Tiangen Biotech Co., Ltd. (Beijing, China).

#### 2.2.2. Rat Model of Myocardial Infarction and Herbs Pretreatment

Male Wistar rats aged 8–10 weeks were obtained from the Experimental Animal Research Center of Inner Mongolia University (Huhehaote, China). The rats were divided into five groups of 10 rats each. The control group rats received 0.3% sodium carboxymethyl cellulose (CMC-Na) solution for a period of 21 days (2 mL/kg/day, i.g.) and normal saline (1 mL/kg) by subcutaneous injection (s.c.) on the 20th and 21st day. Model rats and herb-treated rats each received 0.3% CMC-Na solution/GuangZao/RouDouKou/FCN for a period of 21 days and 85 mg/kg of ISO hydrochloride (ISO (Shanghai, China), 1 mL/kg, s.c.) on the 20th and 21st days to induce myocardial injury, and the doses of three herb-treated groups were 650 mg/kg/day, i.g. All animals received standard diet and water* ad libitum*. The experimental protocols were conducted in accordance with the Guidelines for the Care and Use of Laboratory Animals issued by Inner Mongolia Medical University and approved by the Committee of Animal Ethics.

#### 2.2.3. Electrocardiography

At the end of the experimental period, ECG were recorded by biological data acquisition and analysis system (BL-420F, Techman Soft, China). Under chloral hydrate (400 mg/kg, i.p.) anesthesia, blood was collected via the abdominal aorta. After clotting the blood, the serum was separated by centrifugation at 3,000 rpm, 10 min. The serum and heart tissues were stored at −80°C until further analysis.

#### 2.2.4. Assay of Serum Myocardial Injury Markers

Activity of serum creatine kinase-MB (CK-MB) and lactate dehydrogenase (LDH) was measured using commercial kits (Biosino Bio-Technology and Science, Inc., Beijing, China) with an automated chemical analyzer (Biobase-Sapphire, Ireland).

#### 2.2.5. Infarct Size Determination

The hearts of rats that had been pretreated in different ways were sliced into six sections along the long axis, incubated in 2% TTC [2,3,5-triphenyltetrazolium chloride dissolved in PBS (0.1 mol·L-1)] for 20 min at 37°C. The infarct area (IA) was determined and calculated as described by Zhang [28]. Briefly, images were captured and the area was adjusted for weight. IA/LV% were calculated according to the formulas: IA/LV% = [(W1×I1+ W2×I2+ W3×I3+ W4×I4+ W5×I5+ W6×I6)÷WT]  ×  %, where Wn is the weight of each heart section; WT is the weight of the entire heart; and In is the percentage of infarct area (IA) that was white in color in each section.

#### 2.2.6. Histopathological Analysis

Myocardial tissue was fixed in 10% buffered formalin at least 48 h. Subsequently, the fixed tissues were embedded, sectioned, and stained (H&E). The specimens were examined under a light microscope (DM4000, Leica Germany) by experienced pathologists. Photomicrographs were taken at a magnification of 400×. The histological findings were graded according to the following scoring system [29]: (−) no changes; (+) mild (focal myocytes damage or small multifocal degeneration with slight degree of inflammatory process); (++) moderate (extensive myofibrillar degeneration and/or diffuse inflammatory process); (+++) marked (necrosis with diffuse inflammatory process).

#### 2.2.7. Quantitative Real-Time PCR

qRT-PCR was performed on a Step-One Plus (Applied Biosystems). GAPDH was used to normalize gene expression. The following primer sequences were used:* GAPDH*: forward 5′-CGGCAAGTTCAACGGCACAG-3′, reverse 5′-GACGCCAGTAGACTCCACGACAT-3′;* Bax*: forward 5′-GCAGAGGATGATTGCTGATGTGG-3′, reverse 5′-TCCCGAAGTAGGAAAGGAGGC-3′;* Bcl-2*: forward 5′-TGGCATCTTCTCCTTCCAGCCT-3′, reverse 5′-AGTTCCTCCACCACCGTGGCAAAGC-3′;* IL-1β:* forward 5′-GAGGCTGACAGACCCCAAAA-3′, reverse 5′-GCTCCACGGGCAAGACAT-3′;* TNF-α*: forward 5′-ACAAGGCTGCCCCGACTATG-3′, reverse 5′-CCCGGACTCCGTGATGTCTAA-3′;* p53*: forward 5′-GTCACCTCCACACCTCCACCTG-3′, reverse 5′-TGCCTGTCGTCCAGATACTCAGC-3′; and* p38*: forward 5′-GGGACCTAAAGCCCAGCAAC-3′, reverse 5′-GCATCCAATTCAGCATAATCTCG-3′. Oligo synthesis was performed by Sangon Biotech (Shanghai, China).

#### 2.2.8. Western Blot

The samples were homogenized in RIPA buffer (APPLYGEN) added protease inhibitor (Roche). The suspension was centrifuged for 15 min at 12,000*g* at 4°C. The supernatant was boiled for 10 min in order to denature the proteins. The protein extracts were separated by electrophoresis on 12% SDS-polyacrylamide gels and transferred onto polyvinylidene fluoride (PVDF) membranes (Roche, USA). Membranes were incubated with sealed liquid (5% nonfat milk and 0.01% Tween-20 in TBS) for 1 h, then incubated first overnight with the following primary antibodies: rabbit antibody against p53 (1:1,000; Cell Signaling, #2527), P-p53 (1:1,000; Cell Signaling, #2521), p38 (1:1,000; Cell Signaling, #8690), P-p38 (1:1,000; Cell Signaling, #4511), or *β*-actin (1:1,000; Kangcheng, China). Finally, the membranes were incubated with horseradish peroxidase-conjugated anti-rabbit secondary antibody (Biotime, China). Enhanced chemiluminescent immunoblotting (ECL, Bio-Rad, Japan) was used to visualize the membranes. Imag-Proplus 6.0 software was used to calculate the integrated absorbance (IA) of the bands.

#### 2.2.9. Statistical Analysis

Data are presented as mean ± SD. One-way ANOVA was used to determined statistical differences, and Tukey's test was performed for statistical comparisons among the groups. The S-N-K test was used when there was homogeneity of variance, and the chi-square test was employed to assess incidence.* P*< 0.05 was considered statistically significant.

## 3. Results and Discussion

### 3.1. Establishment of the Network of Anti-CHD Targets of FCN and Topological Analysis

GuangZao (*F. Choerospondiatis), *a widely known Mongolian herb, derived from the dried fruit of* C. axillaris* (Roxb.) Burtt et Hill. Its preparation is described in traditional Mongolian medicinal theory. It has been used extensively as a remedy for ischemic heart disease and has been shown to have good clinical efficacy. It contains of several kinds of useful components, including organic acids, phenolic acids, and flavonoids [8–12]. RouDouKou (*Nutmeg*) is also a valued kitchen spice, but it has been often used for remedies for the stomach, heart, and nerve disorders in traditional Mongolian medicine. RouDouKou is a rich source of fixed and essential oils, triterpenes, and various types of phenolic compounds [13–26]. The essential compounds of GuangZao and RouDouKou are shown in Supplementary Table S1.

Statistical analysis of Mongolian medicine prescriptions for the treatment of cardiovascular disease in “Mongolian Medicine Prescription” and “Medicine Standard (1998 edition)” was conducted using binary quantization (expressed by 1 and 0), and SPSS22.0 was used for cluster analysis. Cluster analysis (Supplementary Figure S1) indicated that, at a distance of 25, the prescription ingredients can be divided into two categories, one for GuangZao* (Fructus Choerospondiatis), *RouDouKou* (Nutmeg), Syzygium aromaticum *(Clove),* Radix aucklandiae*, and* Aquilaria agallocha Roxb. *(Agilawood) and another for other herbs. The highest degree of association was observed between the GuangZao and RouDouKou.

#### 3.1.1. Extracting Active Components in FCN

The present study identified 25 components in GuangZao and 38 in RouDouKou, including those by filtering with DL *⩾*0.18 and OB*⩾*30%; constituents migrating to blood, high-content compounds, and compounds listed as active in previous reports were included for further investigation. Altogether, these compounds accounted for 55.3% of the essential compounds are shown in Supplementary Table S2.

#### 3.1.2. Drug Target Prediction and Synergistic Action Analysis

According to the filters previously described in the methodology, we obtained 453 candidate genes that were targeted by FCN. We found 126 common targets by overlap analysis among GuangZao and RouDouKou to prove that this herb pair acts synergistically. By comparing the candidate targets of FCN to known CHD-related genes obtained from OMIM and DrugBank databases, we found 87 CHD-related genes in the candidate targets of FCN, in which 24 genes frequencies greater than 4 were shown in Supplementary Table S3.

#### 3.1.3. Network of Compound-Target and Analysis

**Figure 2:**
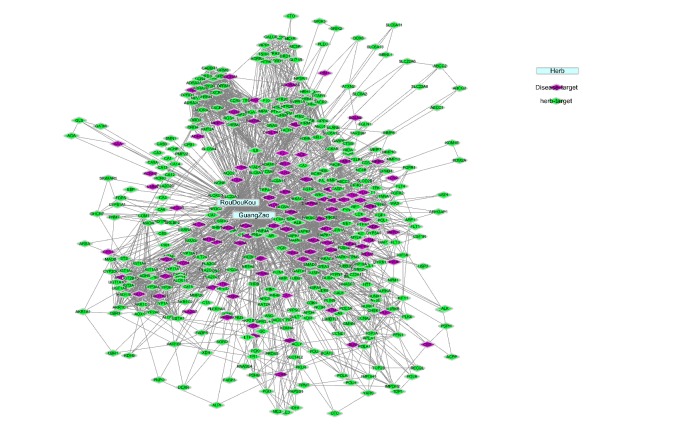
Network of FCN-targets-interacted proteins. The blue nodes represent herbs, the purple nodes represent disease targets, and the green nodes represent predicted targets.

The compound-target network was constructed using Cytoscape 3.5.1 software, which included 2,343 protein interactions between candidate targets (Figure 2). After calculation of topological features, if the* Degree, Betweenness,* and* Closeness* of this node were greater than median of corresponding parameters of all nodes, then the nodes were designated as hub nodes. Our results showed that the hub nodes included 20 key compounds and 29 key targets.

**Figure 3:**
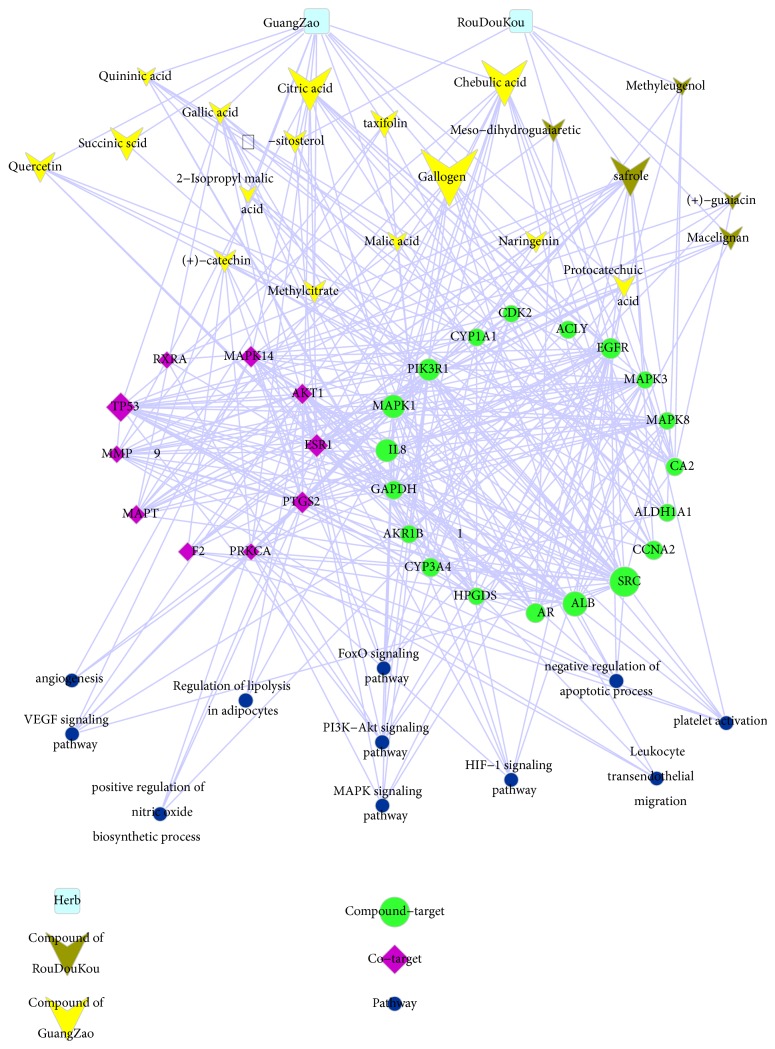
FCN-compound-target-pathway network. The pool blue and square shape nodes represent herbs, the yellow anchor-shaped nodes represent compound of GuangZao, the dark green anchor-shaped nodes represent compound of RouDouKou, the purple rhombus shape nodes represent cotargets (both disease targets and compound targets), the green rhombus nodes represent compound targets, and the dark blue nodes represent pathways.

These 29 key targets included 10 cotargets that are important in the pathogenesis of CHD: mitogen-activated protein kinase 14 (MAPK14/p38), cellular tumor antigen p53 (TP53/p53), serine/threonine-protein kinases 1 (AKT1), prothrombin (F2), and matrix metalloproteinase-9 (MMP9) (Figure 3). The activation of MAPK14 in the course of myocardial ischemia injury aggravates cellular injury induces inflammation, apoptosis, and other cellular processes [30]. In response to ischemia reperfusion (I/R) stress, p53 has been known to become activated and accumulated in the mitochondrial matrix. This gave rise to ROS generation, subsequently causes transient mitochondrial outer membrane permeabilization (MOMP) and the release of cytochrome C, which triggers apoptosis by activating caspase 3 [31]. The action of Akt on its downstream targets determines its function in cardiovascular processes such as cell survival, growth, proliferation, angiogenesis, mediating eNOS phosphorylation, and vasorelaxation [32]. F2 is a common target of antithrombotic drugs, and MMP9 plays an essential role in local proteolysis of the extracellular matrix and in leukocyte migration. The top three in the* Betweenness *order are p53, ESR1, and p38 in the 10 cotargets of FCN (*Betweenness*, N = 0.042509, 0.024799, 0.019129). The detailed information of 10 cotargets is listed in Supplementary Table S4.

The 20 key compounds included 15 GuangZao, 4 RouDouKou ingredients and 1 common composition, indicating that GuangZao plays significant roles against CHD in FCN. Among the ingredients, gallogen and citric acid found in GuangZao and safrole found in RouDouKou exhibited the highest* degree*s (N = 116, 102, 100) to CHD, followed by chebulic acid and gallic acid (*Degree*, N=87, 52) from GuangZao (Figure 3, Supplementary Table S5).

#### 3.1.4. Network of Compound-Target-Pathway and Analysis

Then, based on the results of DAVID analysis, we conducted enrichment analysis using KEGG pathways and GO terms according to key targets of FCN. As is shown in Figure 3, the seven enriched pathways and four GO function terms are closely related to CHD, including the pathways of VEGF signaling pathway, PI3K-Akt signaling pathway, leukocyte transendothelial migration, FoxO signaling pathway, HIF-1 signaling pathway, regulation of lipolysis in adipocytes, MAPK signaling pathway, and the GO terms of negative regulation of apoptotic process, angiogenesis, positive regulation of nitric oxide biosynthetic process, and platelet activation. Acute cardiovascular events, on account of rupture or erosion of an atherosclerotic plaque, are the major cause of morbidity and mortality in patients. Intraplaque (IP) neovascularization and hemorrhage, hypoxia, and influx of inflammatory mediators are stimulating factor of plaque rupture. The molecular mechanism of regulating this pathological process involves the HIF-1 signaling pathway, VEGF signaling pathway, angiogenesis, inflammatory response, leukocyte transendothelial migration, and other processes [33]. Reperfusion therapy is believed to be the most effective method of treating acute myocardial infarction (AMI), but it may lead to further damage of ischemic myocardium, which is related to p38/MAPK activation, myocardial apoptosis, PI3K-Akt signaling cascade, endothelial nitric oxide synthase (eNOS) activity, and immune system activation [34, 35]. These representative pathways indicate that FCN acts as an anti-CHD agent.

### 3.2. Pharmacodynamic Study of FCN

#### 3.2.1. The Effect of FCN on ECG

**Figure 4:**
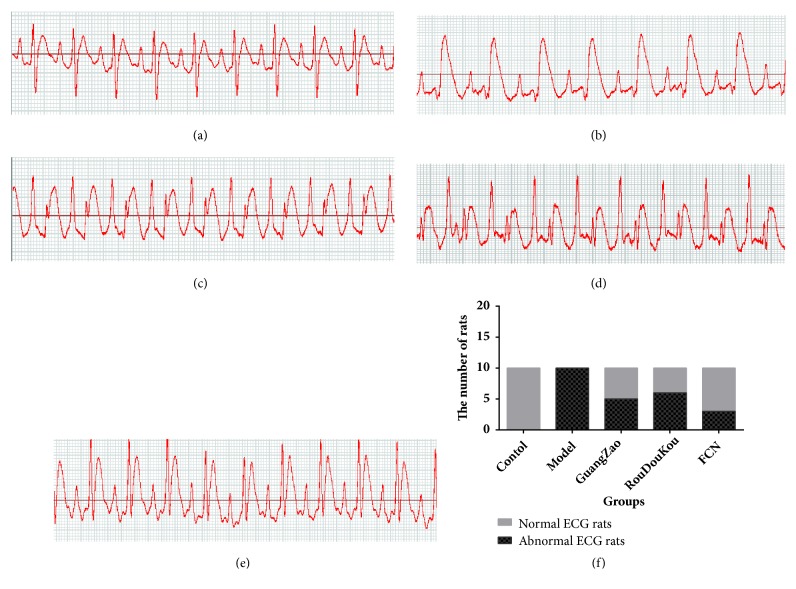
(a-e) Electrocardiographic patterns. (a) ECG of normal rat's heart showing normal ST segments. (b) ECG of ISO-treated rats (85 mg/kg body weight). (c) ECG of GaoZao treated rats (650 mg/kg body weight). (d) ECG of RouDouKou-treated rats (650 mg/kg body weight). (e) ECG of FCN (325 mg/kg GaoZao +325 mg/kg RouDouKou body weight) treated rats showing relatively normal ST segments. (f) The ratio of abnormal ECG rats in each group.

To verify the effect of FCN on myocardial ischemia, we established the rat model of myocardial ischemia. Figures 4(a)–4(e) show the ECG patterns of model rats induced with 85 mg/kg ISO (model group, Figure 4(b)) revealed ST segments upward; the incidence of abnormal ECG showed a significant difference between normal and ISO model group (*χ*^2^ = 5.495,* P *= 0.019,* P *< 0.05). The incidence of abnormal ECG was 50.0% in the GaoZao group and 70% in RouDouKou group (650 mg/kg body weight) (GaoZao and RouDouKou group; Figures 4(c) and 4(d)). The ECG of the major ISO rats pretreated with FCN was generally normal, and the rate of abnormal ECG was 30% (FCN group; Figure 4(e)). Chi-square testing indicated that the difference was statistically significant, with a difference of *χ*^2^=9.295,* P*= 0.002,* P*<0.05.

#### 3.2.2. Serum Cardiac Markers

The levels of CK-MB and LDH in the blood samples from each group were measured. CK-MB and LDH content in the model group was significantly elevated compared to the control group (CK-MB, 44.23 ± 8.22, and 97.16 ± 10.3^##^; LDH, 509.2 ± 37.81 and 837.2 ± 45.14^##^) (control and model group). The CK-MB and LDH levels of the FCN+ISO-treated rats (325 mg/kg GaoZao + 325 mg/kg RouDouKou+85 mg/kg ISO body weight) significantly decreased (*P*<0.05) compared to model rats (CK-MB, 59.23 ± 11.21^*∗∗*^; LDH, 619.2 ± 92.34^*∗*^) (FCN group). However, the levels of CK-MB and LDH from the rats pretreated with GuangZao and RouDouKou did not significantly differ compared to model rats (CK-MB, 65.56 ± 14.76 and 72.45 ± 13.3; LDH, 657.8 ± 65.31 and 673.89 ± 55.19) (GaoZao and RouDouKou group).

#### 3.2.3. TTC Macroscopic Enzyme Mapping Assay

**Figure 5:**
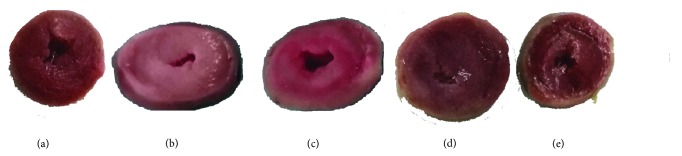
Photographs of representative samples stained by triphenyl tetrazolium chloride (TTC). (a) Normal rat heart. (b) ISO-treated heart exhibits most necrotic areas, which stain a few with TTC. (c) RouDouKou+ ISO rat heart shows roughly 1/3 stain with TTC. (d)-(e) Heart of GuangZao+ ISO and FCN+ISO rats show a few necrotic heart tissue.

To determine the extent of myocardial ischemia, the cardiac tissues were sectioned and stained with TTC. Figures 5(a)–5(e) exhibited that the hearts of model rats depicted a large unstained area, with several necrotic patches (Figure 5(b)); rats pretreated with GuangZao alone or FCN showed more positively stained area indicating tissue viability with less necrosis (Figures 5(d) and 5(e)).

#### 3.2.4. Histological Changes

**Figure 6:**
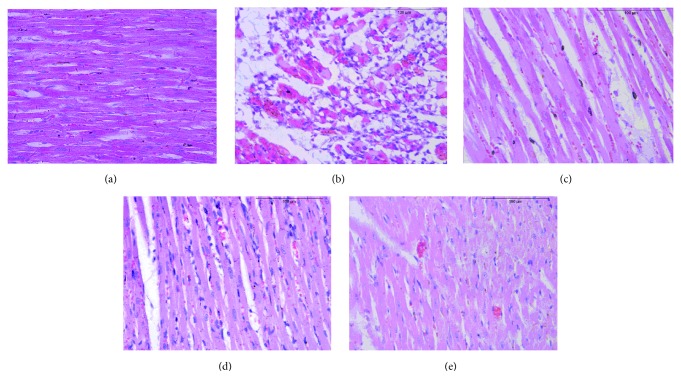
Histopathological observations of the heart (100×). (a) Control rat heart revealed normal architecture of myocardium. (b) ISO-induced myocardial infarcts show cardiac muscle separation, necrosis with inflammatory cells. (c) RouDouKou-treated rats showed a small amount of vacuolar degeneration, and the myocardial fibers have a relatively wider gap. (d) GuangZao group showed evidence of inflammatory infiltration and relatively small gaps between myocardial fibers. (e) FCN group only showed mild separation of cardiac muscle fibers without necrosis and inflammatory cells.

We explored further the effect of FCN in ISO-mediated histological changes of hearts. Figures 6(a)–6(e) show that while the heart tissues of control rats showed normal myocardial fibers, extensive disruption and fragmentation of heart myofibrils with visible necrosis, loss of striations, hyperemia, and inflammatory infiltration were observed in the model rats. Administration of RouDouKou, GuangZao, or FCN all reduced the ISO-induced myocardial necrosis in different degrees. The heart tissue of RouDouKou-treated rats showed a small amount of vacuolar degeneration, and the myocardial fibers showed wider gaps. GuangZao group showed evidence of inflammatory infiltration and relatively narrow gaps among myocardial fibers; meanwhile, FCN group merely displayed a small amount of hemorrhage.

These results suggested that FCN imparts a therapeutic effect on myocardial ischemia.

### 3.3. Experimental Validation of the Network Pharmacology

#### 3.3.1. Quantitative Real-Time PCR

**Figure 7:**
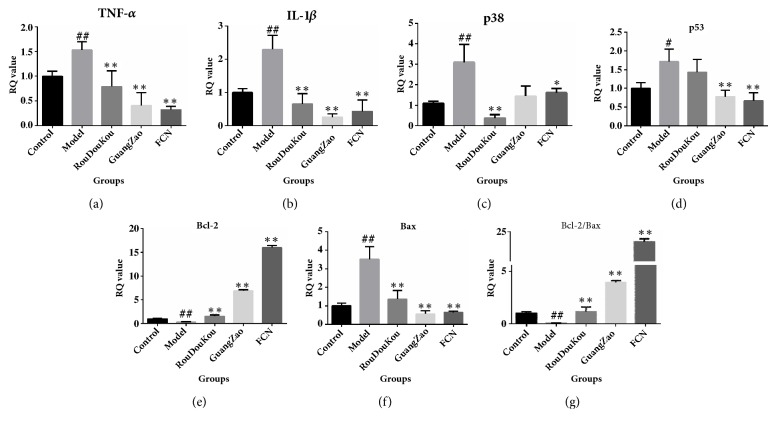
The FCN alters the expression of genes that are related to stress reaction, apoptosis and necrosis of cardiomyocytes, the inflammatory response. (a–f) mRNA levels of the proinflammatory cytokines TNF-*α*, IL-1*β*, stress cytokine p38, and the proapoptosis cytokines P53, Bax, and antiapoptosis cytokines Bcl-2, as well as Bcl-2/Bax ratio of the injured myocardium of each intervention group (n = 6 mice per group). Mean values ± SD. are represented. ^##^*P* < 0.01* versus* the control group, ^#^*P *< 0.05* versus* the control group, *∗∗P* < 0.01 versus model, and *∗P* < 0.05 versus model.

ISO induces multiple histological changes of hearts, including stress reaction, apoptosis, and necrosis of cardiomyocytes, the inflammatory responses, and other similar processes. Previous studies have revealed that the 10 cotargets of FCN included MAPK14 (p38) and TP53 (p53) (*Degree*, N=74, 48) (Supplementary Table S4). To verify the results of network analysis, the gene expression of p38 and p53, the downstream proinflammatory cytokines TNF-*α* and IL-1*β*, and apoptosis-related gene Bcl2 and Bax in each group were detected by qRT-RCR. The levels of p38, p53, TNF-*α*, IL-1*β*, and Bax dramatically increased in the model rat heart tissues compared to the control (*P*<0.01). In addition, Bcl-2 expression significantly decreased (*P*<0.01). Treatment with RouDouKou or GuangZao resulted in the downregulation of p38, p53, TNF-*α*, IL-1*β*, and Bax in different degrees compared with model group. The p38 levels of the RouDouKou group were lower than those of the GuangZao group (*P*<0.05), whereas the p53, TNF-*α*, IL-1*β*, and Bax levels of the GuangZao group were lower than the RouDouKou group. However, Bcl-2 expression and Bcl-2/Bax ratio showed the opposite profiles. The above targets in the FCN group showed similar profiles to those of the RouDouKou or GuangZao groups, and FCN induced more pronounced effects (Figures 7(a)–7(g)).

#### 3.3.2. Western Blot

MAPK14 (p38) plays an important role in the cascades of cellular responses evoked by cell stresses such as proinflammatory cytokines or physical stress, which is activated through phosphorylation on both Thr-180 and Tyr-182 and further phosphorylate additional targets including TP53(p53) [36]. p53 regulates cell-cycle checkpoint and induction of apoptosis in response to DNA damage. The available evidence suggests that promoter selectivity of p53 is regulated by its phosphorylation [37]. For instance, phosphorylation of Ser15 and Ser20 influences binding of p53 to promoters for cell-cycle arrest and DNA repair genes. An additional phosphorylation of Ser46 following severe DNA damage increases the affinity of p53 for promoters of proapoptotic genes. Gallogen (ellagic acid) and quercetin in GuangZao and macelignan in RouDouKou are key compounds which exhibited the higher* degree*s* (Degree*, N=116,44,32). It has been reported that gallogen could exert its beneficial effects by regulating multiple genes, including several cell survival/cell-cycle genes such as p53, Bcl-2, Bax, and c-Myc and regulation of kinases, like MAPK, phosphoinositide 3-kinase (PI3-K), glycogen synthase kinase 3 beta (GSK-3), and so on [38]. Quercetin and macelignan were reported that the protective effects on hepatoprotective effect or anti-inflammatory may be associated with repression of P-p38 [22, 39].

**Figure 8:**
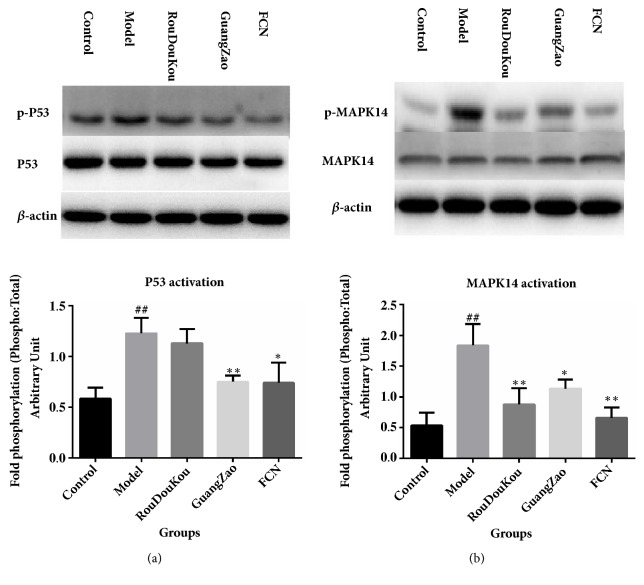
FCN alters p38 and p53 expression in the ISO-induced rats. (a) The protein expression levels of p38 in each group. (b) The protein expression levels of p53 in each group. Groups included FCN, GuangZao, and RouDouKou group (650 mg/kg body weight). Data are expressed as the mean value ± SD (n = 6 mice per group). ^##^*P* < 0.01* versus* control group, *∗∗P* < 0.01* versus* model, and *∗P* < 0.05* versus* model.

To evaluate the actions of FCN on stress reaction and cardiomyocyte apoptosis, the crucial predicted targets, including p38 and p53 protein levels in each group, were analyzed by western blotting. With different treatments, distinct changes in p38 and p53 expression were not observed among the five groups. Under the conditions of existing methods about network analysis and target prediction, whether small molecules affect protein expression or phosphorylation is not yet distinguishable. To further explore the involvement of p38 and p53, P-p38 and P-p53 protein levels in each group were analyzed. We found that FCN, GuangZao, and RouDouKou groups showed significantly lower levels of P-p38 (*P*< 0.01 (FCN, RouDouKou) or* P*< 0.05 (GuangZao), respectively,* versus* model) compared to the model rats (Figure 8(b)). Moreover, the levels of P-p53 in FCN, GuangZao rats were significantly lower (*P* < 0.05 (FCN),* P* < 0.01 (GuangZao),* versus* model) (Figures 8(a) and 8(b)). RouDouKou group showed lower levels of P-p38 expression than GuangZao (*P* < 0.05, RouDouKou* versus* GuangZao), whereas the RouDouKou group expressed lower levels of P-p53 than the RouDouKou (P =0.076, RouDouKou* versus* GuangZao). These findings suggest that FCN treatment effectively reduced stress reactions and cardiomyocyte apoptosis in ISO-induced rats possibly by interfering with phosphorylation levels of p38 or p53, which further affected downstream genes, rather than protein levels of p38 and p53. Overall, the results were consistent with systematic pharmacological analysis.

## 4. Conclusions

FCN, a traditional Mongolian herb pair for CHD treatment in China, can ameliorate ischemia in ISO-induced myocardial ischemia rats. In this study, we developed an integrative systems pharmacology approach to explore the pharmacological mechanisms of FCN against CHD by which a network pharmacology analysis and experimental validation. For the first time, we identified 25 in GuangZao and 38 in RouDouKou candidate compounds and they were predicted to bind to 89 CHD-related targets using multiple online platforms. We further defined the molecular mechanisms of FCN against CHD through multilevel data integration, including a compound-target-interacted proteins network, compound-target- pathway network, and topological analysis. The results confirm that the combination of GuangZao and RouDouKou represses myocardial ischemia in ISO rats by regulating reaction abilities, which in turn inhibits cardiomyocyte apoptosis and inflammatory responses. Results indicate that GuangZao and RouDouKou are effective for the treatment of CHD.

## Data Availability

The data used to support the findings of this study are available from the corresponding author upon request.

## Supplementary Materials

Supplementary Materials Supplementary Table S 1-1: compounds of GuangZao. Supplementary Table S 1-2: compounds of RouDouKou. Supplementary Table S 2-1: candidate compounds of GuangZao. Supplementary Table S 2-2: candidate compounds of RouDouKou. Supplementary Table S3: the potential targets of compounds of FCN on CHD (frequency ≥ 4). Supplementary Table S4: the key targets of FCN (ordered by “Betweenness” from large to small). Supplementary Table S5: the key compounds of FCN (ordered by “Degree” from large to small).
