# Supplementary material for: Understanding the Multitarget Pharmacological Mechanism of the Traditional Mongolian Common Herb Pair GuangZao-RouDouKou Acting on Coronary Heart Disease Based on a Bioinformatics Approach
Source: Evid Based Complement Alternat Med. 2018 Nov 11;2018:7956503. doi: 10.1155/2018/7956503 (PMC6252196; doi:10.1155/2018/7956503)
Supplement: Supplementary Materials — Supplementary Table S 1-1: compounds of GuangZao. Supplementary Table S 1-2: compounds of RouDouKou. Supplementary Table S 2-1: candidate compounds of GuangZao. Supplementary Table S 2-2: candidate compounds of RouDouKou. Supplementary Table S3: the potential targets of compounds of FCN on CHD (frequency ≥ 4). Supplementary Table S4: the key targets of FCN (ordered by “Betweenness” from large to small). Supplementary Table S5: the key compounds of FCN (ordered by “Degree” from large to small). [file 7956503.f1.zip › 7956503.f1/Supplementery Materials/Supplementary Table S3 The potential targets of compounds of FCN on CHD .docx]

Supplementary Table S3: The potential targets of compounds of FCN on CHD (frequency≥4)

| Name | Target | Frequency | Overlapped herbs |
| --- | --- | --- | --- |
| Estrogen receptor | ESR1 | 19 | GuangZao, RouDouKou |
| Transthyretin | TTR | 18 | GuangZao, RouDouKou |
| Microtubule-associated protein tau | MAPT | 16 | GuangZao, RouDouKou |
| Mitogen-activated protein kinase 14 | MAPK14 | 16 | GuangZao, RouDouKou |
| Prostaglandin G/H synthase 2 | PTGS2 | 10 | GuangZao, RouDouKou |
| Liver carboxylesterase 1 | CES1 | 8 | GuangZao, RouDouKou |
| Glucocorticoid receptor | NR3C1 | 8 | GuangZao, RouDouKou |
| Apolipoprotein A-II | APOA2 | 7 | RouDouKou |
| 3-hydroxy-3-methylglutaryl-coenzyme A reductase | HMGCR | 7 | GuangZao, RouDouKou |
| Nuclear factor erythroid 2-related factor 2 | NFE2L2 | 7 | GuangZao, RouDouKou |
| Estrogen receptor beta | ESR2 | 6 | GuangZao, RouDouKou |
| Prelamin-A/C | LMNA | 6 | GuangZao, RouDouKou |
| Serine/threonine-protein kinase pim-1 | PIM1 | 6 | GuangZao, RouDouKou |
| Peroxisome proliferator-activated receptor gamma | PPARG | 6 | GuangZao, RouDouKou |
| Prostaglandin G/H synthase 1 | PTGS1 | 6 | GuangZao, RouDouKou |
| Bloom syndrome protein | BLM | 5 | GuangZao |
| Cytochrome P450 2C19 | CYP2C19 | 5 | GuangZao, RouDouKou |
| Cytochrome P450 2C9 | CYP2C9 | 5 | GuangZao, RouDouKou |
| Tyrosine-protein phosphatase non-receptor type 11 | PTPN11 | 5 | GuangZao, RouDouKou |
| Angiotensin-converting enzyme 2 | ACE | 4 | GuangZao |
| Arachidonate 5-lipoxygenase | ALOX5 | 4 | GuangZao, RouDouKou |
| L-lactate dehydrogenase B chain | LDHB | 4 | GuangZao |
| Macrophage migration inhibitory factor | MIF | 4 | GuangZao |
| 72 kDa type IV collagenase | MMP2 | 4 | GuangZao |
| Matrix metalloproteinase-9 | MMP9 | 4 | GuangZao, RouDouKou |
| cAMP-specific 3',5'-cyclic phosphodiesterase 4D | PDE4D | 4 | GuangZao, RouDouKou |
| Retinoic acid receptor RXR-alpha | RXRA | 4 | GuangZao, RouDouKou |
