# Supplementary material for: Understanding the Multitarget Pharmacological Mechanism of the Traditional Mongolian Common Herb Pair GuangZao-RouDouKou Acting on Coronary Heart Disease Based on a Bioinformatics Approach
Source: Evid Based Complement Alternat Med. 2018 Nov 11;2018:7956503. doi: 10.1155/2018/7956503 (PMC6252196; doi:10.1155/2018/7956503)
Supplement: Supplementary Materials — Supplementary Table S 1-1: compounds of GuangZao. Supplementary Table S 1-2: compounds of RouDouKou. Supplementary Table S 2-1: candidate compounds of GuangZao. Supplementary Table S 2-2: candidate compounds of RouDouKou. Supplementary Table S3: the potential targets of compounds of FCN on CHD (frequency ≥ 4). Supplementary Table S4: the key targets of FCN (ordered by “Betweenness” from large to small). Supplementary Table S5: the key compounds of FCN (ordered by “Degree” from large to small). [file 7956503.f1.zip › 7956503.f1/Supplementery Materials/Supplementary Table S 1 GangZao and RouDouKou Compound.doc]

Supplementary Table S 1-1: Compounds of GuangZao

| Codes | Compound | Molecular formula | CAS/CID | Kinds |
| --- | --- | --- | --- | --- |
| gz1 | Malic Acid | C_4_H_6_O_5_ | 617-48-1 | organic acids |
| gz2 | 2-Isopropyl malic acid | C_7_H_12_O_5_ | [49601-06-1](https://www.chemsrc.com/cas/49601-06-1_401121.html) | organic acids |
| gz3 | Citric Acid | C_6_H_8_O_7_ | 77-92-9 | organic acids |
| gz4 | Succinic Acid | C_4_H_6_O_4_ | 110-15-6 | organic acids |
| gz5 | Trimethyl citrate | C_9_H_14_O_7_ | 1587-20-8 | organic acids |
| gz6 | Triethyl citrate | C_12_H_20_O_7_ | 77-93-0 | organic acids |
| gz7 | Monoglyceride citrate | C_9_H_14_O_9_ | 109350-12-1 | organic acids |
| gz8 | Stearic acid | C_18_H_36_O_2_ | 57-11-4 | organic acids |
| gz9 | Ursolic acid | C_30_H_48_O_3_ | 77-52-1 | organic acids |
| gz10 | Quininic acid | C_11_H_9_NO_3_ | 86-68-0 | phenolic acids |
| gz11 | (-)-Chebulic acid | C_14_H_12_O_11_ | 27739-95-3 | phenolic acids |
| gz12 | Gallic acid | C_7_H_6_O_5_ | 149-91-7 | phenolic acids |
| gz13 | Protocatechuic acid | C_7_H_6_O_4_^-^ | 99-50-3 | phenolic acids |
| gz14 | Gallogen /Ellagic acid | C_14_H_6_O_8_ | 476-66-4 | phenolic acids |
| gz15 | 3,7,8-Tri-O-methylellagic acid 4-glucoside | C_23_H_22_O_13_ | 114942-09-5 | phenolic acids |
| gz16 | 3,3'-Di-O-methylellagic acid | C_16_H_10_O_8_ | 2239-88-5 | phenolic acids |
| gz17 | 3-O-methylellagic acid | C_15_H_8_O_8_ | 13915428 | phenolic acids |
| gz18 | Bis(2-ethylhexyl) phthalate | C_24_H_38_O_4_ | 117-81-7 | phenolic acids |
| gz19 | Ethyl gallate | C_24_H_38_O_4_ | 831-61-8 | phenolic acids |
| gz20 | 3,8-Di-O-methylellagic acid 2-O-glucoside | C_22_H_20_O_13_ | 51803-68-0 | phenolic acids |
| gz21 | Dibutyl phthalate | C_16_H_20_O_4_ | 84-74-2 | phenolic acids |
| gz22 | 3-Hydroxybenzoic acid | C_7_H_6_O_3_ | 99-06-9 | phenolic acids |
| gz23 | P-Hydroxybenzoic acid | C_7_H_6_O_3_ | 99-96-7 | phenolic acids |
| gz24 | Salicylic acid | C_7_H_6_O_3_ | 69-72-7 | phenolic acids |
| gz25 | Comenic acid | C_6_H_4_O_5_ | 499-78-5 | phenolic acids |
| gz26 | (+)-catechin | C_15_H_14_O_6_ | 154-23-4 | flavonoids |
| gz27 | (-)-catechin | C_15_H_14_O_6_ | 18829-70-4 | flavonoids |
| gz28 | Quercetin | C_15_H_10_O_7_^-^ | 117-39-5 | flavonoids |
| gz29 | Taxifolin | C_15_H_12_O_7_ | 480-18-2 | Flavonoids |
| gz30 | Naringenin | C_15_H_12_O_5_ | 67604-48-2 | flavonoids |
| gz31 | Dimeric catechin | C_30_H_24_O_12_ | 44460468 | flavonoids |
| gz32 | Hyperin | C_21_H_19_O_12_ | 90657624 | flavonoids |
| gz33 | Quercetin 3-rhamnoside | C_21_H_20_O_11_ | 522-12-3 | Flavonoids |
| gz34 | Quercetin-3-arabinopyranoside | C_20_H_18_O_11_ | 22255-13-6 | flavonoids |
| gz35 | Tyrosine | C_9_H_11_NO_3_ | 60-18-4 | amino acids |
| gz36 | Proline | C_5_H_9_NO_2_ | 147-85-3 | amino acids |
| gz37 | Isoleucine | C_6_H_13_NO_2_ | 73-32-5 | amino acids |
| gz38 | Leucine | C_6_H_13_NO_2_ | 61-90-5 | amino acids |
| gz39 | Phenylalanine | C_9_H_11_NO_2_ | 63-91-2 | amino acids |
| gz40 | β-sitosterol | C_29_H_50_O | 83-46-5 | others |
| gz41 | 5-hydroxymethyl furaldehyde（5-HMF) | C_6_H_6_O_3_ | 67-47-0 | others |
| Gz42 | Pantothenic acid | C_9_H_17_NO_5_ | 79-83-4 | others |
| Gz43 | Protocatechualdehyde | C_7_H_6_O_3_ | 139-85-5 | others |
| gz44 | Scopoletin | C_10_H_8_O_4_ | 92-61-5 | others |

Supplementary Table S 1-2: Compounds of RouDouKou

| Codes | Compound | Molecular formula | CAS/CID | Kinds |
| --- | --- | --- | --- | --- |
| rdk1 | α-pinene | C_10_H_6_ | 80-56-8 | monoterpenoids |
| rdk2 | 4-terpineol | C_10_H_18_O | 562-74-3 | monoterpenoids |
| rdk3 | γ-terpinene | C_10_H_16_ | 99-85-4 | monoterpenoids |
| rdk4 | （+）-limonene | C_10_H_16_ | 5989-27-5 | monoterpenoids |
| rdk5 | （-）-limonene | C_10_H_16_ | 5989-54-8 | monoterpenoids |
| rdk6 | sabinene | C_10_H_16_ | 3387-41-5 | monoterpenoids |
| rdk7 | α-terpineol | C_10_H_18_O | 98-55-5 | monoterpenoids |
| rdk8 | α-terpinene | C_10_H_16_ | 99-86-5 | monoterpenoids |
| rdk9 | β-pinene | C_10_H_16_ | 127-91-3 | monoterpenoids |
| rdk10 | β-caryophyllene | C_15_H_24_ | 87-44-5 | monoterpenoids |
| rdk11 | β-phellandrene | C_10_H_16_ | 555-10-2 | monoterpenoids |
| rdk12 | myristicin | C_11_H_12_O_3_ | 607-91-0 | phenylpropanoids |
| rdk13 | elemicin | C_12_H_16_O_3_ | 487-11-6 | phenylpropanoids |
| rdk14 | methyleugenol | C_11_H_14_O_2_ | 93-15-2 | phenylpropanoids |
| rdk15 | safrole | C_10_H_10_O_2_ | 94-59-7 | phenylpropanoids |
| rdk16 | eugenol | C_10_H_12_O_2_ | 97-53-0 | phenylpropanoids |
| rdk17 | Methylisoeugenol | C_11_H_14_O_2_ | 93-16-3 | phenylpropanoids |
| rdk18 | p-cymene | C_11_H_14_ | 99-87-6 | phenylpropanoids |
| rdk19 | carvacrol | C_10_H_14_ O | 499-75-2 | phenylpropanoids |
| rdk20 | Isoeugenol | C_10_H_12_O_2_ | 97-54-1 | phenylpropanoids |
| rdk21 | dehydrodiisoeugenol | C_20_H_22_O_4_ | 2680-81-1 | phenylpropanoids |
| rdk22 | tetrahydrofuroguaiacin B | C_20_H_24_O_5_ | 13870572 | lignans |
| rdk23 | saucernetindiol | C_20_H_24_O_5_ | 40580526 | lignans |
| rdk24 | (+)-Verrucosin | C_20_H_24_O_5_ | 10736226 | lignans |
| rdk25 | nectandrin A | C_21_H_26_O_5_ | 13939326 | lignans |
| rdk26 | nectandrin B | C_20_H_24_O_5_ | 74683-16-2 | lignans |
| rdk27 | fragransin C1 | C_21_H_26_O_6_ | 13870579 | lignans |
| rdk28 | All-trans-7,7'-Epoxylignan | C_22_H_26_O_6_ | 102445801 | lignans |
| rdk29 | galbacin | C_20_H_20_O_5_ | 234441 | lignans |
| rdk30 | macelignan | C_20_H_24_O_4_ | 107534-93-0 | lignans |
| rdk31 | 1,1-Diphenylbutane | C_16_H_18_ | 719-79-9 | lignans |
| rdk32 | Hydroxyachillin | C_15_H_18_O_4_ | 35879-92-6 | lignans |
| rdk33 | Meso-monomethyldihydroguaiaretic acid | C_21_H_28_O_4_ | 11725068 | lignans |
| rdk34 | 1,1-Diphenylbutane | C_16_H_18_ | 719-79-9 | lignans |
| rdk35 | (+)-guaiacin | C_20_H_24_O_4_ | 36531-08-5 | lignans |
| rdk36 | All-trans-7,7'-Epoxylignan | C_22_H_26_O_6_ | 67604-48-2 | lignans |
| rdk37 | Meso-dihydroguaiaretic acid | C_20_H_26_O_4_ | 36469-60-0 | lignans |
| rdk38 | Machilin A | C_20_H_22_O_4_ | 10359012 | lignans |
| rdk39 | Machilin D | C_20_H_24_O_4_ | 5936610 | lignans |
| rdk40 | Lignans machilin F | C_20_H_22_O_4_ | 13844301 | lignans |
| rdk41 | myristargenol B | C_20_H_26_O_5_ | 26195071 | lignans |
| rdk42 | argenteane | C_40_H_46_O_8_ | 131752652 | lignans |
| rdk43 | otobaphenol | C_20_H_22_O_4_ | 10240-16-1 | lignans |
| rdk44 | Licarin A | C_20_H_22_O_4_ | 2680-81-1 | new lignans |
| rdk45 | licarin C | C_20_H_26_O_5_ | 131751410 | new lignans |
| rdk46 | licarin B | C_20_H_20_O_4_ | 51020-87-2 | new lignans |
| rdk47 | myrifralignan A | C_21_H_24_O_6_ | 101902714 | new lignans |
| rdk48 | myrifralignan B | C_24_H_28_O_8_ | 101902715 | new lignans |
| rdk49 | myrifralignan C | C_21_H_26_O_6_ | 21770239 | new lignans |
| rdk50 | myrifralignan D | C_22_H_26_O_7_ | 25751146 | new lignans |
| rdk51 | myrifralignan E | C_22_H_26_O_8_ | 101902716 | new lignans |
| rdk52 | myrislignan | C_21_H_26_O_6_ | 21636106 | new lignans |
| rdk53 | erythro-(7S,8R)-∆8′-7-acetoxy-3,4,3′,5′,-tetramethoxyphenyl)-8-O-4′-neolignan | C_24_H_29_O_7_ |  | new lignans |
| rdk54 | erythro-2-（4-allyl-2，6-dimethoxyphenoxy)-1-(3,4,5-trimethoxyphenyl)-propane | C_22_H_29_O_6_ |  | new lignans |
| rdk55 | (?)-Raphidecursinol B | C_23_H_30_O_7_ | 25751148 | new lignans |
| rdk56 | Surinamensin | C_22_H_28_O_6_ | 5281868 | new lignans |
| rdk57 | Myrisfrageal A | C_18_H_16_O_5_ | 101663168 | new lignans |
| rdk58 | Myrisfrageal B | C_19_H_18_O_6_ | 102225414 | new lignans |
| rdk59 | Isoeugenol acetate | C_12_H_14_O_3_ | 1715136 | new lignans |
| rdk60 | malabaricone B | C_21_H_26_O_4_ | 63335-24-0 | malabaricones |
| rdk61 | Malabaricone C | C_21_H_26_O_5_ | 63335-25-1 | malabaricones |
| rdk62 | malabaricone A | C_21_H_26_O_3_ | 63335-23-9 | malabaricones |
| rdk63 | ((8R,8′S)-7-(4-hydroxy-3-methoxyphenyl)-8′-methylbutan-8-yl)-3′-methoxybenzene-4′,5′-diol | C_17_H_24_O_5_ |  | malabaricones |
| rdk64 | Diphenylpropane | C_15_H_16_ | 1530-03-6 | Phenyl alkanes |
| rdk65 | 2-Methoxy-4-[(E)-prop-1-enyl]benzene-1,3,5-triol | C_10_H_12_O_4_ | 129704203 | Phenylpropanediol |
| rdk66 | Dihydrodihydroxy-safrole | C_10_H_12_O_4_ | 129677021 | Phenylpropanediol |
| rdk67 | 2-Dodecylcyclobutanone | C_16_H_30_O | 35493-46-0 | alkylcyclobutanones |
| rdk68 | 2-decylcyclobutanone | C_14_H_26_O | 15311392 | alkylcyclobutanones |
| rdk69 | β-sitosterol | C_29_H_50_O | 83-46-5 | others |
| Rdk70 | daucosterol | C_35_H_60_O_6_ | 474-58-8 | others |
