# Supplementary material for: Understanding the Multitarget Pharmacological Mechanism of the Traditional Mongolian Common Herb Pair GuangZao-RouDouKou Acting on Coronary Heart Disease Based on a Bioinformatics Approach
Source: Evid Based Complement Alternat Med. 2018 Nov 11;2018:7956503. doi: 10.1155/2018/7956503 (PMC6252196; doi:10.1155/2018/7956503)
Supplement: Supplementary Materials — Supplementary Table S 1-1: compounds of GuangZao. Supplementary Table S 1-2: compounds of RouDouKou. Supplementary Table S 2-1: candidate compounds of GuangZao. Supplementary Table S 2-2: candidate compounds of RouDouKou. Supplementary Table S3: the potential targets of compounds of FCN on CHD (frequency ≥ 4). Supplementary Table S4: the key targets of FCN (ordered by “Betweenness” from large to small). Supplementary Table S5: the key compounds of FCN (ordered by “Degree” from large to small). [file 7956503.f1.zip › 7956503.f1/Supplementery Materials/Supplementary Table S 2 Candidate compounds of GangZao and RouDouKou.doc]

Supplementary Table S 2-1: Candidate compounds of GuangZao

| Codes | Compound | Structure | CAS/CID | OB | DL | Screening basis | Literatures |
| --- | --- | --- | --- | --- | --- | --- | --- |
| gz1 | Malic acid |  | 617-48-1 | 59.62 | 0.02 | Constituents migrating to blood | 7, 8 |
| gz2 | 2-Isopropyl malic acid |  | [49601-06-1](https://www.chemsrc.com/cas/49601-06-1_401121.html) |  |  | Constituents migrating to blood | 7, 8 |
| gz3 | Citric acid |  | 77-92-9 | 56.22 | 0.05 | Constituents migrating to blood, around 15% of GangZao water extract | 7, 8, 9 |
| gz4 | Succinic scid |  | 110-15-6 | 29.62 | 0.62 | Constituents migrating to blood | 7, 8 |
| gz5 | Trimethyl citrate |  | 1587-20-8 | 67.61 | 0.07 | Constituents migrating to blood | 7, 8 |
| gz6 | Triethyl citrate |  | 77-93-0 |  |  | Constituents migrating to blood | 7 |
| gz10 | Quininic acid |  | 86-68-0 | 63.53 | 0.06 | Constituents migrating to blood | 7, 8 |
| gz11 | (-)-Chebulic acid |  | 27739-95-3 | 72 | 0.32 | Constituents migrating to blood | 7, 8 |
| gz12 | Gallic acid |  | 149-91-7 | 31.69 | 0.04 | Constituents migrating to blood , around 0.08% of GangZao water extract | 7, 8, 9 |
| gz12m | 4-O-Methylgallic acid |  |  | 36.74 | 0.05 | The metabolite of Gallic acid | 7 |
| gz13 | Protocatechuic acid |  | 99-50-3 | 25.37 | 0.04 | Constituents migrating to blood, around 0.13% of GangZao water extract | 7, 8, 9 |
| gz14 | Gallogen/ellagic acid |  | 476-66-4 | 43.06 | 0.43 | Constituents migrating to blood | 7, 8 |
| gz16 | 3,3'-Di-O-methylellagic acid |  | 2239-88-5 | 23.53 | 0.53 | Constituents migrating to blood | 7 |
| gz17 | 3-O-methylellagic acid |  | 13915428 |  |  | Constituents migrating to blood | 7 |
| gz18 | Bis(2-ethylhexyl) phthalate |  | 117-81-7 | 43.59 | 0.35 | eligibility (OB≥30%；DL ≥0.18) | TCMSP |
| gz13m | vanillic acid |  | 121-34-6 | 35.47 | 0.04 | The metabolite, the methylate of Protocatechuic acid | 7 |
| gzm1 | P-methylphenol |  | 106-44-5 | 53.95 | 0.01 | The metabolite of phenolic acids and flavonoids | 7 |
| gzm2 | m-methylphenol |  | 108-39-4 | 51.99 | 0.01 | The metabolite of phenolic acids and flavonoids | 7 |
| gz26 | (+)-catechin |  | 154-23-4 | 54.84 | 0.24 | Constituents migrating to blood | 7, 8 |
| gz27 | (-)-catechin |  | 18829-70-4 | 49.68 | 0.24 | Constituents migrating to blood | 7, 8 |
| gz28 | Quercetin |  | 117-39-5 | 46.43 | 0.28 | Constituents migrating to blood， around 0.0003% of GangZao water extract， glycosylated aglycone | 7, 8, 9 |
| gz29 | taxifolin |  | 480-18-2 | 57.84 | 0.27 | Eligibility (OB≥30%；DL ≥0.18) | TCMSP, 10 |
| gz30 | Naringenin |  | 67604-48-2 | 59.29 | 0.21 | Eligibility (OB≥30%；DL ≥0.18) | TCMSP |
| gz40 | β-sitosterol |  | 83-46-5 | 36.91 | 0.75 | Eligibility (OB≥30%；DL ≥0.18) | TCMSP, 11 |
| gz41 | 5-hydroxymethyl furaldehyde（5-HMF) |  | 67-47-0 | 45.07 | 0.02 | Constituents migrating to blood | TCMSP , 12 |

Supplementary Table S 2-2: Candidate compounds of RouDouKou

| Codes | Compound | Structure | CAS/CID | OB | DL | Screening basis | Literatures |
| --- | --- | --- | --- | --- | --- | --- | --- |
| rdk1 | α-pinene |  | 80-56-8 | 46.25 | 0.05 | High content, anti-microbial, antioxidant | 13,21,24,TTD |
| rdk2 | 4-terpineol |  | 562-74-3 | 81.41 | 0.03 | High content, constituents migrating to blood | 13,22 |
| rdk3 | γ-terpinene |  | 99-85-4 | 33.02 | 0.02 | High content | 13,TTD |
| rdk4 | （+）-limonene |  | 5989-27-5 | 39.84 | 0.02 | High content | 13 |
| rdk5 | （-）-limonene |  | 5989-54-8 | 38.09 | 0.02 | High content | 13 |
| rdk6 | sabinene |  | 3387-41-5 | 20.48 | 0.08 | High content | 13,24 |
| rdk7 | α-terpineol |  | 98-55-5 | 48.8 | 0.03 | High content | 13 |
| rdk8 | α-terpinene |  | 99-86-5 | 33.95 | 0.02 | High content | 13,TTD |
| rdk9 | β-pinene |  | 127-91-3 | 44.84 | 0.05 | High content, anti-microbial, antioxidant | 13,21,22,TTD |
| rdk11 | β-phellandrene |  | 555-10-2 | 40.44 | 0.02 | High content | 24 |
| rdk12 | myristicin |  | 607-91-0 | 17.99 | 0.07 | High content, toxicant, anti-microbial, antioxidant, constituents migrating to blood | 13,21,22,24,TTD |
| rdk13 | elemicin |  | 487-11-6 | 21.94 | 0.06 | High content | 13,20,TTD |
| rdk14 | methyleugenol |  | 93-15-2 | 73.36 | 0.04 | High content | 13,TTD |
| rdk15 | safrole |  | 94-59-7 | 34.21 | 0.06 | High content, constituents migrating to blood | 13,22,24,TTD |
| rdk16 | eugenol |  | 97-53-0 | 56.24 | 0.04 | High content | 13,TTD |
| rdk17 | methylisoeugenol |  | 93-16-3 | 32.61 | 0.04 | High content | 13,TTD |
| rdk19 | carvacrol |  | 499-75-2 | 43.28 | 0.03 | High content, anti-microbial, antioxidant | 21 |
| rdk20 | isoeugenol |  | 97-54-1 | 70.1 | 0.04 | High content High content, | 21 |
| rdk21 | Dehydrodiisoeugenol |  | 2680-81-1 | 56.84 | 0.29 | Anti-inflammatory inhibiting NO, anti-inflammatory | 19, 26 |
| rdk22 | tetrahydrofuroguaiacin B |  | 13870572 | 62.86 | 0.32 | Activating AMPK, had effect to metabolic syndrome, such as type 2 diabete | 13,23 |
| rdk23 | Saucernetindiol |  | 40580526 | 41.85 | 0.32 |  | 13, TCMSP |
| rdk24 | (+)-Verrucosin |  | 10736226 | 4.09 | 0.32 | Hypermeability through the blood-brain barrier | 13, 25 |
| rdk25 | nectandrin A |  | 13939326 |  |  | Activating AMPK | 14, 24 |
| rdk26 | nectandrin B |  | 74683-16-2 | 4.09 | 0.32 | Activating AMPK, hypermeability through the blood-brain barrier | 13, 23, 25 |
| rdk29 | galbacin |  | 234441 | 61 | 0.53 | Anti-inflammatory, suppress MAPK, hepatoprotective effect | 13 |
| rdk30 | macelignan |  | 107534-93-0 | 23.61 | 0.32 | Anti-inflammatory, suppress MAPK, hepatoprotective effect | 13, 16, 17, 18, 22 |
| rdk35 | (+)-guaiacin |  | 36531-08-5 | 48.78 | 0.31 | Activating AMPK, had effect to metabolic syndrome, such as type 2 diabete | 13, 23, 25 |
| rdk37 | meso-dihydroguaiaretic acid |  | 36469-60-0 | 1.34 | 0.21 | Anti-microbial | 13,25 |
| rdk38 | machilin A |  | 10359012 | 10.56 | 0.29 | Activating AMPK | 13 |
| rdk44 | licarin A |  | 2680-81-1 | 9.75 | 0.31 | Anti-inflammatory, anti-microbial | 13, TTD |
| rdk46 | licarin B |  | 51020-87-2 | 11.35 | 0.4 | Anti-sunburn | 13 |
| rdk57 | Myrisfrageal A |  | 101663168 |  |  | Inhibiting NO, anti-inflammatory | 19 |
| rdk58 | Myrisfrageal B |  | 102225414 |  |  | Inhibiting NO, anti-inflammatory | 19 |
| rdk52 | myrislignan |  | 21636106 |  |  | Anti-inflammatory, inhibiting CYP2C9, hypermeability through the blood-brain barrier | 13, 14, 15, 25 |
| rdk60 | malabaricone B |  | 63335-24-0 | 1.04 | 0.35 | Bioactivity | 13, 25 , TCMSP |
| rdk61 | malabaricone C |  | 63335-25-1 | 1.04 | 0.35 | Treating vascular disease, repairing gastric ulcer, anti-infection，promoting angiogenesis, antioxidant, anti-microbial, anti-cancer | 13, 14 |
| rdk63 | ((8R,8′S)-7-(4-hydroxy-3-methoxyphenyl)-8′-methylbutan-8-yl)-3′-methoxybenzene-4′,5′-diol |  |  |  |  | Anti-inflammatory, inhibiting NO, | 14 |
| rdk69 | β-sitosterol |  | 83-46-5 | 36.91 | 0.75 |  | 13 |
|  |  |  |  |  |  |  |  |
