# Supplementary material for: Understanding the Multitarget Pharmacological Mechanism of the Traditional Mongolian Common Herb Pair GuangZao-RouDouKou Acting on Coronary Heart Disease Based on a Bioinformatics Approach
Source: Evid Based Complement Alternat Med. 2018 Nov 11;2018:7956503. doi: 10.1155/2018/7956503 (PMC6252196; doi:10.1155/2018/7956503)
Supplement: Supplementary Materials — Supplementary Table S 1-1: compounds of GuangZao. Supplementary Table S 1-2: compounds of RouDouKou. Supplementary Table S 2-1: candidate compounds of GuangZao. Supplementary Table S 2-2: candidate compounds of RouDouKou. Supplementary Table S3: the potential targets of compounds of FCN on CHD (frequency ≥ 4). Supplementary Table S4: the key targets of FCN (ordered by “Betweenness” from large to small). Supplementary Table S5: the key compounds of FCN (ordered by “Degree” from large to small). [file 7956503.f1.zip › 7956503.f1/Supplementery Materials/Supplementary Table S4.doc]

Supplementary Table S4: The key targets of FCN (ordered by “Betweenness” from large to small)

| Name | Targets | Betweenness | Closeness | Degree | Property of targets |
| --- | --- | --- | --- | --- | --- |
| Proto-oncogene tyrosine-protein kinase Src | SRC | 0.043677 | 0.482633 | 59 | compound-target |
| Tumor protein p53 | TP53 | 0.042509 | 0.47482 | 74 | co-target |
| Serum albumin | ALB | 0.029877 | 0.453219 | 49 | compound-target |
| Interleukin-8 | IL8 | 0.025539 | 0.428224 | 51 | compound-target |
| Estrogen receptor | ESR1 | 0.024799 | 0.471008 | 44 | co-target |
| Mitogen-activated protein kinase 1 | MAPK1 | 0.023653 | 0.473543 | 61 | compound-target |
| Mitogen-activated protein kinase 14 | MAPK14 | 0.019129 | 0.436003 | 48 | co-target |
| Phosphatidylinositol 3-kinase regulatory subunit alpha | PIK3R1 | 0.018977 | 0.412178 | 52 | compound-target |
| Prostaglandin G/H synthase 2 | PTGS2 | 0.018963 | 0.453219 | 42 | co-target |
| Epidermal growth factor receptor | EGFR | 0.01622 | 0.458732 | 48 | compound-target |
| Androgen receptor | AR | 0.015024 | 0.438903 | 38 | compound-target |
| Microtubule-associated protein tau | MAPT | 0.014194 | 0.444071 | 26 | co-target |
| serine/threonine-protein kinases 1 | AKT1 | 0.014123 | 0.442953 | 48 | co-target |
| Cyclin-A2 | CCNA2 | 0.014078 | 0.433498 | 28 | compound-target |
| Cytochrome P450 3A4 | CYP3A4 | 0.013733 | 0.416404 | 42 | compound-target |
| Glyceraldehyde-3-phosphate dehydrogenase | GAPDH | 0.012049 | 0.443697 | 37 | compound-target |
| Prothrombin | F2 | 0.011752 | 0.412823 | 38 | co-target |
| Carbonic anhydrase 2 | CA2 | 0.011176 | 0.437448 | 18 | compound-target |
| Aldose reductase | AKR1B1 | 0.011168 | 0.402746 | 18 | compound-target |
| ATP-citrate synthase | ACLY | 0.01049 | 0.385401 | 24 | compound-target |
| Retinal dehydrogenase 1 | ALDH1A1 | 0.009942 | 0.40678 | 26 | compound-target |
| Cytochrome P450 1A1 | CYP1A1 | 0.00987 | 0.40553 | 37 | compound-target |
| Matrix metalloproteinase-9 | MMP9 | 0.009178 | 0.425121 | 32 | co-target |
| Mitogen-activated protein kinase 8 | MAPK8 | 0.009046 | 0.423416 | 45 | compound-target |
| Hematopoietic prostaglandin D synthase | HPGDS | 0.008609 | 0.421389 | 31 | compound-target |
| Retinoic acid receptor RXR-alpha | RXRA | 0.008553 | 0.392565 | 23 | co-target |
| Peroxisome proliferator-activated receptor gamma | PPARG | 0.007979 | 0.394914 | 21 | co-target |
| Cyclin-dependent kinase 2 | CDK2 | 0.007704 | 0.399093 | 33 | compound-target |
| Kinesin-like protein KIF11 | KIF11 | 0.00766 | 0.373409 | 25 | compound-target |
