# Supplementary material for: Understanding the Multitarget Pharmacological Mechanism of the Traditional Mongolian Common Herb Pair GuangZao-RouDouKou Acting on Coronary Heart Disease Based on a Bioinformatics Approach
Source: Evid Based Complement Alternat Med. 2018 Nov 11;2018:7956503. doi: 10.1155/2018/7956503 (PMC6252196; doi:10.1155/2018/7956503)
Supplement: Supplementary Materials — Supplementary Table S 1-1: compounds of GuangZao. Supplementary Table S 1-2: compounds of RouDouKou. Supplementary Table S 2-1: candidate compounds of GuangZao. Supplementary Table S 2-2: candidate compounds of RouDouKou. Supplementary Table S3: the potential targets of compounds of FCN on CHD (frequency ≥ 4). Supplementary Table S4: the key targets of FCN (ordered by “Betweenness” from large to small). Supplementary Table S5: the key compounds of FCN (ordered by “Degree” from large to small). [file 7956503.f1.zip › 7956503.f1/Supplementery Materials/Supplementary Table S5.doc]

Supplementary Table S5: The key compounds of FCN (ordered by “Degree” from large to small)

| Compounds | Betweenness | Closeness | Degree | Herbs |
| --- | --- | --- | --- | --- |
| Gallogen | 0.122302 | 0.464789 | 116 | GuangZao |
| Citric acid | 0.084946 | 0.45478 | 102 | GuangZao |
| safrole | 0.074345 | 0.452442 | 100 | RouDouKou |
| Chebulic acid | 0.089725 | 0.461942 | 87 | GuangZao |
| Gallic acid | 0.0231 | 0.424779 | 52 | GuangZao |
| taxifolin | 0.036783 | 0.410895 | 51 | GuangZao |
| (+)-catechin | 0.023255 | 0.418052 | 46 | GuangZao |
| Quercetin | 0.044177 | 0.404288 | 44 | GuangZao |
| Protocatechuic acid | 0.021827 | 0.398491 | 41 | GuangZao |
| Succinic scid | 0.05305 | 0.375533 | 40 | GuangZao |
| Meso-dihydroguaiaretic | 0.024871 | 0.394619 | 38 | GuangZao |
| Methylcitrate | 0.02433 | 0.383442 | 37 | GuangZao |
| β-sitosterol | 0.024137 | 0.39789 | 33 | GuangZao, RouDouKou |
| Macelignan | 0.026306 | 0.380403 | 32 | RouDouKou |
| Malic acid | 0.01461 | 0.38013 | 29 | GuangZao |
| Naringenin | 0.020185 | 0.382886 | 25 | GuangZao |
| 3,3'-Di-O-methylellagic acid | 0.005338 | 0.37931 | 24 | GuangZao |
| Methyleugenol | 0.011195 | 0.371569 | 23 | RouDouKou |
| (+)-guaiacin | 0.008233 | 0.365651 | 22 | RouDouKou |
| Quininic acid | 0.011433 | 0.385683 | 21 | GuangZao |
